# Knowledge, Attitude, and Practice towards Influenza Vaccination among Older Adults in Southern China during the COVID-19 Pandemic

**DOI:** 10.3390/vaccines11071197

**Published:** 2023-07-04

**Authors:** Yating You, Xiaoheng Li, Binglin Chen, Xuan Zou, Gang Liu, Xinxin Han

**Affiliations:** 1 School of Public Health and Emergency Management, Southern University of Science and Technology, Shenzhen 518055, China; 2 Shenzhen Center for Disease Prevention and Control, Shenzhen 518055, China

**Keywords:** influenza, vaccine, older adults, KAP, COVID-19

## Abstract

Influenza is prevalent globally, leading to severe morbidity and mortality. During the pandemic, knowledge, attitude, and practice (KAP) towards influenza virus and vaccination were less investigated among southern Chinese older adults. A cross-sectional study was conducted through the structured questionnaire among community healthcare centers in selected districts in Shenzhen, southern China from September to October 2021. KAP towards influenza virus and vaccination were analyzed. A multivariable logistic regression model was used to identify associated factors. Among 975 participants, 55.6% were reported to have received influenza vaccination ever, and 46.6% had taken influenza vaccination in 2020 during the pandemic. Only one-fifth of participants knew severe comorbidities happen among severe influenza cases. A total of 88.3% thought older adults should have influenza vaccination. COVID-19 vaccination history was associated with receiving influenza vaccination (OR 1.92, 95% CI 1.32–2.80). People with a high-level income had better KAP towards influenza virus and vaccination. COVID-19 vaccination history was associated with the positive actions of influenza vaccination during the pandemic. Efforts should be made to promote the free influenza vaccination program widely and launch health education events on influenza and its vaccination regularly to improve KAP among older adults.

## 1. Introduction

Influenza is prevalent globally, leading to severe morbidity and mortality [1,2]. It is estimated that worldwide influenza causes approximately 3 to 5 million cases of severe illness and up to 650,000 respiratory deaths every year [2]. Older adults and people with chronic physical conditions are deemed to be at an increased risk of infection and hospitalization due to influenza [2]. In China, it was estimated that roughly 80% of the excess respiratory deaths attributed to influenza were among people aged 60 years or above between 2010 and 2015 [1,3]. With the evolution of the circulating influenza virus, annual vaccination is recommended as the most effective tool to protect against influenza, as immunity is not lifelong [4,5]. WHO recommends the composition of the trivalent vaccine that targets the three most representative virus types in circulation (two subtypes of influenza A viruses and one influenza B virus) [2]. Hence, launching the influenza vaccination program among older adults is a significant approach to alleviating the influenza disease burden.

With the implementation of national medical reform in China in 2009, vaccination services were provided through community health centers (CHCs), including vaccines for influenza, hepatitis B, human papillomavirus, COVID-19, etc. [6,7]. People of all ages can have influenza vaccine uptakes with their out-of-pocket money of 11 to 49 US dollars (76–334 Chinese Yuan) yearly at widely distributed CHCs in urban areas. During the COVID-19 period, since March 2021, the Chinese government has started to offer the COVID-19 vaccine to older adults for free after domestically comprehensive assessments of health status and infection risks [8]. Although the State Council encouraged local governments to offer free vaccination services to highly-risky populations, including healthcare professionals, children aged between 6 months and 5 years, people with chronic diseases, and others who take care of vulnerable populations, free influenza vaccination services were limited and provided only for older adults in some developed Chinese provinces and cities, for example, Zhejiang Province, Beijing, Guangzhou, as well as our study site, Shenzhen City [9]. Despite many conveniences, compared with influenza vaccination coverage in developed countries, 82.3% in England, 90.2% in Scotland, 75.2% in the United States, 55.0% in Russia, and 56.8% in France during the 2022 flu season, in China, according to previous research, it remained low (4–13%) among older adults [10,11,12,13].

Previous studies focused on the knowledge, attitude, and practice (KAP) of healthcare professionals, pregnant women, travelers, and general workers towards influenza virus and vaccination in different countries and regions [14,15,16,17]. A satisfactory level of knowledge related to COVID-19 and its prevention among the Italian elderly was found [18], which promised a positive attitude and practice towards vaccination. Evidence showed that vaccination-prioritized populations including older adults having insufficient knowledge were associated with low vaccination coverage [19], which indicated that lack of knowledge had an impact on their attitude and practice of vaccination. However, related evidence on the KAP of vaccination is quite limited in China. For example, one study focusing on older adults aged 60 years and above in eastern China was conducted in 2015 to describe early and regional KAP results related to influenza and its vaccination [12]. Less is known about KAP towards influenza virus and vaccination among older adults in southern China during the COVID-19 period with the appearance of free vaccination services.

This study aimed to investigate KAP towards influenza virus and vaccination among 975 Chinese older adults aged 60 years and above and identify associated influenza-related factors with their actions of vaccination. In addition, our study would provide valuable insight into the potential target population and suggest corresponding measures to improve vaccination coverage more efficiently, which is beneficial to promote vaccination-related policy. 

## 2. Materials and Methods

### 2.1. Study Design and Participants

From 24 September to 20 October 2021, a cross-sectional survey was conducted on the demographic and socioeconomic information, disease and vaccination history, and KAP regarding influenza virus and vaccination in Shenzhen, China. Shenzhen is located in southern China, being one of the first-tier cities with more than 17 million residents. There are 10 districts with more than 700 community health centers in Shenzhen. Ten CHCs in two large districts (Nanshan and Guangming) of Shenzhen were randomly selected as our study sites.

Participants were included through convenient sampling in our study if they (1) were aged 60 years old and above before September 2021; (2) attended the above-included CHCs; and (3) were willing to complete the questionnaire. Considering the feasibility of the study design and the strong mobility of the population in the mega city of Shenzhen, as many participants were included as possible. Overall, 993 older adults were enrolled in our study. Of them, 975 participants had complete information and were valid for data analyses. A total of 18 participants were excluded because they did not state their influenza vaccination status, which was conducted as our primary outcome.

### 2.2. Data Collection

Trained interviewers administered the structured questionnaire to included participants, asked questions from the questionnaire to each participant, and wrote down the answers in person. Before this questionnaire was made available to all participants, a pilot study was conducted on a small sample of older participants (N = 30) to ensure that the questionnaire was easily understandable and completed. The structured questionnaire consisted of detailed questions on demographic and socioeconomic characteristics, health conditions, KAP on influenza and its vaccination, as well as health education on vaccination. 

Demographic and socioeconomic characteristics were measured by sex (female and male), age (60–64, 65–75, and above 75 years old), education level (primary school and below, middle and high school, and college and above), marital status (unmarried and married), and monthly income (1000 and below Chinese Yuan (CNY), 1001–5000, and 5001 and above). Health conditions were assessed by self-awareness of health status (well, general, and worse), chronic disease history (yes and no), and COVID-19 vaccination history (yes and no). 

The KAP questionnaire was a part of the structured questionnaire, including knowledge, attitude, and practice towards influenza virus and vaccine. The knowledge survey included influenza virus and vaccine items that were measured by influenza symptoms, prevention measures, vaccination-prioritized populations, vaccination frequencies, vaccination season, vaccine protection duration, the places to get vaccinated, and if they ever knew about influenza vaccine and vaccination policy. The attitude survey included opinions on whether older people and people with chronic diseases should have influenza vaccine uptake and thoughts about vaccination education. The practice survey included cues to actions, vaccine actions, and reasons for being unwilling to receive influenza vaccines. Cues to action consisted of recommendations from healthcare workers, previous vaccination awareness of people around, and participation in vaccine education programs. Actions included self-reported influenza vaccination history before the interview time and in the specific 2020 influenza season. Participants being stratified by influenza vaccination history at that time were categorized as vaccinated status and unvaccinated status as our primary outcome. In addition, participants stratified by influenza vaccination history in the 2020 influenza season were categorized as vaccinated status and unvaccinated status in 2020. 

### 2.3. Statistical Analysis

Statistical analysis was performed with Stata version 17 (StataCorp Inc., Chicago, IL, USA). All categorical variables were described with frequencies (N) and percentages (%). Frequencies and proportions were used to describe the detailed information on knowledge of the influenza virus and vaccine (9 survey items), attitude towards influenza vaccination and its education program (3 survey items), and practice of influenza vaccination (6 survey items). The Chi-square test was used to evaluate the differences in knowledge, attitude, and practice towards influenza virus and vaccination between subgroups, with analyses stratified by age groups, education levels, and income levels. Statistically significant survey items among age groups, education levels, and income levels were included in the following multivariable logistic regression model to identify influenza-associated factors. Demographic and socioeconomic factors stratified by previous actions of influenza vaccine with a *p* value < 0.05 in the Chi-square test were further included in the same multivariable logistic regression model, where fully adjusted odds ratios (ORs) and their corresponding 95% CI were calculated after controlling for other covariates and associated factors. 

Subgroup analysis was conducted among participants who reported as vaccinated in 2020. Statistically significant survey items among age groups, education levels, and income levels were included in the following multivariable logistic regression model to identify influenza-associated factors. Demographic factors stratified by ever received actions of influenza vaccine in 2020 with a *p* value < 0.05 in the Chi-square test were further included in the same multivariable logistic regression model, where ORs and 95% CIs were calculated. A two-sided *p* < 0.05 was considered statistically significant in multivariate analyses.

### 2.4. Ethical Statement

Our study was approved by the Shenzhen Center for Disease Control and Prevention. Oral informed consent was obtained from each participant before interviewers administered and distributed the structured questionnaire.

## 3. Results

### 3.1. Characteristics of Participants

A total of 975 older participants were included in this analysis. In Table 1, it can be seen that more females (56.3%) than males (43.7%) were involved in this analysis. The mean age of participants was 69.4 years old, with a standard deviation of 6.4 years old, and 575 (59%) were between 65 and 75 years old. In total, 823 (87.4%) were married and more than half of the participants had middle and high school diplomas, followed by primary school and below. Overall, 429 (44.0%) had middle-income levels of 1000 to 5000 Chinese yuan (CNY), 592 (60.7%) considered their health well and 441 were diagnosed with chronic medical conditions by a doctor before, and 813 (83.4%) had received COVID-19 vaccine uptakes since the breakout of COVID-19 at the beginning of 2020.

### 3.2. Knowledge of Influenza Virus and Vaccination

In Table 2, only one-fifth of participants (N = 214) knew severe comorbidities would happen among severe cases affected by the influenza virus. Most participants knew about vaccination as the approach to protecting against influenza. In total, 75.6% thought people aged 60 years old and above, children aged 6 months to 5 years old, and patients with chronic conditions were the prioritized populations to take influenza vaccine uptakes, but only 13.1% knew that women who were pregnant or planned to become pregnant during flu season should take influenza vaccination. Overall, 64.2% participants knew the influenza vaccination frequency (once a year), 58.4% knew the influenza vaccination time frame (in autumn and winter), 58.4% knew the protection duration (six to eight months), and 74.1% knew the places to take influenza vaccination. Over 70% of participants have heard about influenza vaccination and knew about the free influenza vaccination program in Shenzhen.

Most older people knew about vaccination as the method to prevent the influenza virus, the protection duration of influenza vaccine, and the places to take vaccination (*p* < 0.05 in three survey items in Table 3). Compared with participants with primary school or above education, participants with higher education levels had better knowledge of influenza symptoms, prioritized populations, vaccination frequency, places, protection duration, as well as vaccination policy (*p* < 0.05 for most survey items in Table 3). So did people who have higher monthly income (*p* < 0.05 for most survey items). The differences in vaccine protection duration and places existed in each age group, educational level, and income level (*p* < 0.05 in three subgroups).

### 3.3. Attitude towards Influenza Vaccination and Education Program

In Table 4, 861 (88.3%) participants thought older adults should take influenza vaccination and over half considered that older adults with chronic medical conditions were bound to take the vaccination. A total of 652 (65.9%) thought it beneficial to participate in the vaccination education program.

Most participants (58.9%) between 65 and 74 years old thought older adults diagnosed with chronic conditions should take influenza vaccination compared with the other two age groups (20.9% vs. 58.9% vs. 20.2%, *p* = 0.010 in Table 5). People with middle and high school education or middle level of income had a more positive attitude towards influenza vaccination and education programs, showing more agreement in three survey items (*p* < 0.05 in Table 5). The differences in attitudes towards vaccination among older adults with chronic diseases existed in each age group, educational level, and income level (*p* < 0.05 in three subgroups).

### 3.4. Practices of Influenza Vaccination

In Table 6, cues to actions of influenza vaccination included three items as follows: 653 (67.0%) participants were recommended the influenza vaccination by healthcare workers, but 542 (55.6%) reported to have received influenza vaccination and 454 (46.6%) have taken influenza vaccine uptakes in 2020 during the COVID-19 period. Around 60% of participants heard about people around who have taken influenza uptakes. One-third of participants have ever taken part in health education programs. In Table 7, additionally, the associations of vaccinated people around previous vaccination status and vaccination status in 2020 differed by age, education, and income levels (*p* < 0.05). Participants among 65 to 74 years old, holding middle and high school diplomas, or with higher income levels, were reported to have received influenza vaccination and taken influenza vaccine uptakes in 2020 (*p* < 0.05).

### 3.5. Reasons for Being Unwilling to Receive the Influenza Vaccines

Among 433 participants who reported not receiving influenza vaccination before, their reasons for being not vaccinated are presented in Table 6. The top three reasons were as follows: participants reported that they did not know about influenza vaccination (43.0%); they thought they stayed in good conditions and there was no need to take the influenza vaccine (27.5%); and they had no time to take the influenza vaccine (12.5%). It is worth noticing that only 2.8% of participants thought the influenza vaccine had no effect. Only 24 (5.5%) participants did not have the influenza vaccine because physicians have not ever recommended it, and another 23 (5.3%) considered it too much cost.

### 3.6. Multivariate Analysis

Using the actions of influenza vaccination as the primary outcome, a multivariate analysis was conducted to determine what variables were associated with our primary outcome. The differences in influenza vaccination status existed in knowledge items of influenza vaccination protective duration, the places to have influenza uptakes, and attitudes towards whether older adults with chronic conditions should take influenza vaccination. In Table 8, compared with younger participants less than 65 years old, participants aged above 65 years old were at higher odds of being vaccinated, with the odds ratio of 1.46 (95% CI 1.02–2.10) for the 65–75 age group and the odds ratio of 1.81 (95% CI 1.10–2.98) for the above 75 age group. Marital status and education levels were not associated with influenza vaccinated status after adjusting for other significant influenza-associated factors. Participants who had a high-level monthly income were associated with higher odds of being vaccinated (5001 and above CNY: OR 2.64, 95% CI 1.66–4.18). COVID-19 vaccination history was not statistically significantly associated with our primary outcome of previous vaccination status before the year 2020. Participants who answered correctly on vaccination protection duration were associated with having influenza vaccine uptakes (OR 2.76, 95% CI 1.99–3.84). Participants who knew about the places of vaccination were associated with the vaccinated status (OR 5.00, 95% CI 3.42–7.32). Moreover, participants who thought older adults with chronic conditions should take influenza vaccination were associated with having vaccination (OR 2.08, 95% CI 1.51–2.85).

### 3.7. Subgroup Analysis

In subgroup analysis, another multivariate regression model stratified by the actions of the influenza vaccine in 2020 was conducted. Identified influenza-associated factors were similar to the results stratified by previous actions of influenza vaccination, but during the COVID-19 period, COVID-19 vaccination history was associated with receiving influenza vaccine uptakes (OR 1.57, 95% CI 1.05–2.37). Participants who had a high-level monthly income were associated with higher odds of being vaccinated (5001 and above CNY: OR 1.69, 95% CI 1.10–2.61). Participants who answered correctly on vaccination protection duration were associated with having influenza vaccine uptakes (OR 2.15, 95% CI 1.58–2.93). Participants who knew about the places of vaccination were associated with the vaccinated status (OR 4.22, 95% CI 2.85–6.24). Moreover, participants who thought older adults with chronic conditions should take influenza vaccination were associated with having vaccination (OR 1.73, 95% CI 1.27–2.35).

## 4. Discussion

A cross-sectional survey was conducted to evaluate the knowledge, attitudes, and practices toward influenza virus and vaccination among 975 Chinese older adults aged 60 years and above and identify influenza vaccination-associated factors from September to October 2021 in southern China. The vaccination coverage was 55.6% among our participants. People with a higher education or income level had a better performance of knowledge, attitude, and practice towards influenza virus and vaccination. People with higher income levels and better knowledge and attitude were associated with the positive actions of influenza vaccination. 

As a result of complex and diverse vaccination policies being implemented in different cities in China, the vaccination coverage rate showed differences among older populations, for example, 55.6% in this study, being much higher than those of previous studies among older adults, ranging from 4.3% to 40.0% [20], but lower than that of the US in 2022 [21]. This difference might be attributed to the fact that people living in Shenzhen are provided with fruitful medical resources and health information offerings from the high-quality primary healthcare system supported by the municipal government [22]. Additionally, evidence showed that health literacy was apparently higher among people in Shenzhen compared with the Chinese average level, which referred to the ability and basic diathesis to acquire and use health information and make health-associated associations, for example, vaccination in the present study [23,24]. Statistically significant influenza-associated factors in knowledge and attitude survey items were identified, which showed that people having sufficient and correct knowledge as well as positive attitude towards influenza vaccination were associated with having vaccination. However, four-fifths of participants were not aware of the occurrence of severe comorbidity, which made people look down upon influenza. A large proportion of them did not know pregnant women as one of the risky populations of infection, which potentially poses a threat to pregnant women and their babies. This reflected the knowledge barriers among our participants. Appropriate health education programs are urgently needed among older adults. 

Previous studies focused on healthcare professionals, pregnant women, and travelers in different regions with the impact of vaccination policies [14,16,17]. Evidence showed that an appropriate vaccination policy indeed has a positive impact on the coverage rate [25], which indicated that a free vaccination policy might attract more attention. The study site, Shenzhen, as the first-tier city in China, has already provided free influenza vaccination among older permanent residents aged 60 years or above for 7 years. Previous studies also proved a positive influence on influenza vaccination coverage under the launch of free influenza vaccination programs for older adults [26]. Vaccination policy was considered one of the most significant vaccination-related factors [27]. Our study also affirmed this factor, stating that older adults who ever knew about the free vaccination program were associated with having the influenza vaccine uptake. This beneficial policy should be generalized among other capable cities to improve influenza vaccination coverage among older adults.

Almost 90% of participants agreed that older adults should take influenza vaccination, but only half of our participants had influenza vaccine uptakes before. The older participants who were less educated were associated with not having influenza vaccination in this study compared with participants with higher education levels. It is consistent with previous studies, indicating that people with low literacy might ignore written information [28], for example, leaflets and posters on free influenza vaccination in CHCs. However, older participants with higher education or income levels in this study knew more about older adults who had influenza vaccination before. It is possible that they were influenced to have vaccine uptakes by their acquaintances, that is, the acquaintance effect. This factor needs to be explored in vaccination studies further. 

The popularity of influenza vaccination was limited to some degree because almost half of the unvaccinated participants reported they did not know about the vaccination program as the top reason. This indicated that the influenza vaccination program should strengthen promotion among the public mass. A small proportion of people considered themselves healthy enough for it to be unnecessary to have influenza uptakes, which was the second reason. This demonstrated the misunderstanding of susceptible populations regarding influenza virus and the necessity of vaccination education programs widely and regularly. Despite the shortages of vaccination programs, a few people thought the influenza vaccine had no effect, which indicated that vaccination education played an important role in improving vaccine-associated knowledge. Several participants mentioned that they were not recommended to have influenza vaccine uptakes by their physicians, but recommending vaccination was supposed to be their work responsibility in our previous studies [26]. This reflected deficiencies in physicians’ work to some degree.

COVID-19 vaccination history was found to be associated with influenza vaccination in 2020, which might be due to their good level of perception on both viruses and their vaccines, being consistent with a cross-sectional study focusing on the knowledge related to COVID-19 among the Italian elderly [18]. During the 2020 flu season with the pandemic, many people perceived the COVID-19 virus as the influenza virus mistakenly because of their similar symptoms, and people were willing to have influenza vaccination when there was no COVID-19 vaccine approved. Our findings suggested that participants with higher education and income levels are associated with having influenza vaccine uptake. Moreover, health and vaccine information on COVID-19 was gradually promoted widely and deeply during the pandemic [29]. The promotion of influenza education cannot reach the point of COVID-19, obviously. Nowadays, with the decreasing cases of COVID-19 daily, the new cases of type A influenza have started to increase and the positive rate is up to 25.1% [30]. Hence, it is urgent to promote influenza and influenza vaccination-related information in China, especially for older adults.

Physicians were reported as a significant factor in multiple studies [15,31]. In our study, similarly, 67% of participants were recommended the influenza vaccination by healthcare workers, and only a few participants stated their un-vaccination status was due to no recommendation from professionals, but half of them were influenza vaccinated. Hence, providing recommendations from professionals is the most convenient and reliable method to improve the influenza vaccination-associated information as well as coverage rate. However, vaccine hesitancy existed among both patients and healthcare professionals. Patients worry about the inefficacy of vaccines and the fear of adverse effects [32]. Professionals may have concerns about the responsibility of medical accidents if an allergy reaction or severe side effects appear after patients’ follow the recommendation of vaccination from them, which needs further investigation as to the reason for not recommending vaccination. Hence, to eliminate vaccine hesitancy, proper and correct vaccination education is needed among healthcare professionals and patients regularly.

Several limitations of this study must be acknowledged. First, recall bias might exist when responding to past experiences. For example, previous vaccination status as our primary outcome was self-reported. Second, considering that the sample was from Shenzhen, where adults aged 60 and above take the proportion of 5.36% (0.95 million), compared with the national average of 18.7% [33,34], our study results might not be generalized to other Chinese cities. Moreover, Shenzhen, as one of four first-tier super cities with the top gross national product in China, is capable of offering financial support for multiple vaccination programs for specific populations. Hence, other governments should tailor their vaccination policy. Third, selection bias might exist, as only participants who visited the included CHCs were included. Fourth, because of the nature of study design, this survey was conducted at the beginning of the influenza season and the attitudes and practices only reflected the information available at a certain point. The actual KAP may have changed after our study period. Future study is needed to assess the KAP towards influenza virus and vaccination among risky populations after the COVID-19 pandemic for further vaccination policy making.

## 5. Conclusions

In conclusion, our study provided valuable insights into the free influenza vaccination policy, which played a significant role in improving vaccination coverage. A relatively high vaccination coverage was found among older adults in Shenzhen. People with higher income levels had a better performance of knowledge, attitude, and practice towards influenza virus and vaccination. COVID-19 vaccination history was associated with the actions of influenza vaccination during the pandemic. Therefore, more actions from the government should be taken to improve influenza vaccination coverage among older adults through providing health education regularly to improve knowledge and attitudes and delivering wide promotion of the free influenza vaccination program to facilitate older adults’ practices.

## Figures and Tables

**Table 1:** Characteristics of study participants in Shenzhen, September 2021 (N = 975).

Characteristics	N	Percentage (%)
Sex		
Female	549	56.3
Male	426	43.7
Age (yrs)		
60–64	230	23.6
65–75	575	59.0
Above 75	170	17.4
Marital status		
Unmarried	152	15.6
Married	823	87.4
Education level		
Primary school and below	338	34.7
Middle and high school	503	51.6
College and above	134	13.7
Monthly income (Chinese Yuan, CNY)		
Low (1000 and below)	266	27.3
Middle (1001–5000)	429	44.0
High (5001 and above)	280	28.7
Self-reported health status		
Well	592	60.7
General	316	32.4
Worse	67	6.9
Chronic medical conditions		
Yes	441	45.2
No	534	54.8
Ever received COVID-19 vaccine uptakes		
Yes	813	83.4
No	162	16.6

**Table 2:** Knowledge of the influenza virus and vaccine among older adults in Shenzhen (N = 975).

Items	N	Percentage (%)
**Influenza virus**		
Q1. What are influenza’s symptoms?		
Acute fever (up to 39–40 Celsius degree)	611	62.7
Vomit, diarrhea	251	25.7
Sore joints, fatigue, decreasing appetite	426	43.7
Runny nose, stuffy nose, sneezing	811	83.2
Serious comorbidity will occur in severe cases	214	22.0
Q2. What is the approach to protecting against influenza?		
Vaccination	717	73.5
Good hygiene	666	68.3
Enhance physical fitness and immunity	655	67.2
Minimize activities in crowded places	461	47.3
If you have respiratory infection symptoms, you should rest at home and seek medical treatment as soon as possible	473	48.5
**Influenza vaccine**		
^a^ Q3. Who are the priority populations that need to take influenza vaccination?		
Healthcare workers	694	71.2
Vulnerable groups and employees	670	68.7
People in key places	424	43.5
Risky populations	737	75.6
Caregivers of infants	266	27.3
Pregnant women	128	13.1
^b^ Q4. Do you know how the influenza vaccination frequency is scheduled?		
Once a year	626	64.2
Every 5 years	349	35.8
Have no idea		
^c^ Q5. Do you know when to get the influenza vaccine?		
In autumn and winter	569	58.4
All year round	406	41.6
Have no idea		
^d^ Q6. How long does the flu vaccine protect after a single dose?		
6–8 months	424	43.5
At least 5 years	551	56.5
Have no idea		
Q7. Do you know where you can get the flu vaccine for seniors?		
Yes	722	74.1
No	253	25.9
Q8. Have you ever heard about influenza vaccination for older adults?		
Yes	759	77.9
No	216	22.1
Q9. Do you know the policy that older adults aged 60 years and above with Shenzhen household registration could have free influenza vaccination in Shenzhen?		
Yes	738	75.7
No	237	24.3

^a^ The details of answers for “Q3. Who are the prioritypopulations that need to take influenza vaccination?” are shown below: healthcare workers, including clinicians, public health staff, and health and quarantine staff; vulnerable groups and employees include people in places where people gather, such as elderly care institutions, long-term care institutions, and welfare homes; people in key places include nursery institutions, primary and secondary schools, and prison institutions; risky populations include older people aged 60 and above, children aged 6 months to 5 years, and patients with specific chronic diseases; infants’ caregivers include family members and caregivers of infants aged under 6 months; pregnant women include women who are pregnant or planning to become pregnant during flu season. ^b^ “Once a year” is the correct answer for Q4. ^c^ “In autumn and winter” is the correct answer for Q5. ^d^ “6–8 months” is the correct answer for Q6.

**Table 3:** Knowledge of the influenza virus and vaccine in the age, education, and income group analysis (N = 975) in Shenzhen, China ^f^.

Items	Age (Yrs)			^a^ *p* Value	Education Level			^a^ *p* Value	Income Level			^a^ *p* Value
	60–64 (N = 230)	65–75 (N = 575)	Above 75 (N = 170)		Primary and Below (N = 338)	Middle and High School (N = 503)	College and Above (N = 134)		Low (N = 266)	Middle (N = 429)	High (N = 280)	
**Influenza virus**												
Q1. What are influenza’s symptoms?												
Acute fever	142 (23.2)	361 (59.1)	108 (17.7)	0.931	192 (31.4)	323 (52.9)	96 (15.7)	0.006	146 (23.9)	268 (43.9)	197 (32.2)	0.001
Vomit, diarrhea	59 (23.5)	152 (60.6)	40 (15.9)	0.748	69 (27.5)	139 (55.4)	43 (17.1)	0.012	51 (20.3)	102 (40.6)	98 (39.0)	<0.001
Sore joints, fatigue, decreasing appetite	108 (25.4)	251 (58.9)	67 (15.7)	0.323	143 (33.6)	217 (50.9)	66 (15.5)	0.366	111 (26.1)	168 (39.4)	147 (34.5)	0.002
Runny nose, stuffy nose, sneezing	195 (24)	472 (58.2)	144 (17.8)	0.550	297 (36.6)	410 (50.6)	104 (12.8)	0.010	225 (27.7)	357 (44.0)	229 (28.2)	0.682
Serious comorbidity	43 (20.1)	131 (61.2)	40 (18.7)	0.386	53 (24.8)	114 (53.3)	47 (22)	<0.001	43 (20.1)	85 (39.7)	86 (40.2)	<0.001
Q2. What is the approach to protecting against influenza?												
Vaccination	151 (21.1)	431 (60.1)	135 (18.8)	0.004	234 (32.6)	374 (52.2)	109 (15.2)	0.022	165 (23.0)	310 (43.2)	242 (33.8)	<0.001
Good hygiene	157 (23.6)	400 (60.1)	109 (16.4)	0.407	213 (32)	355 (53.3)	98 (14.7)	0.030	151 (22.7)	297 (44.6)	218 (32.7)	<0.001
Enhance physical fitness and immunity	145 (22.1)	393 (60)	117 (17.9)	0.309	218 (33.3)	336 (51.3)	101 (15.4)	0.074	156 (23.8)	280 (42.7)	219 (33.4)	<0.001
Minimize activities in crowded places	102 (22.1)	274 (59.4)	85 (18.4)	0.514	148 (32.1)	238 (51.6)	75 (16.3)	0.057	98 (21.3)	196 (42.5)	167 (36.2)	<0.001
Rest at home and seek medical treatment	113 (23.9)	278 (58.8)	82 (17.3)	0.977	157 (33.2)	247 (52.2)	69 (14.6)	0.570	110 (23.3)	202 (42.7)	161 (34.0)	0.001
**Influenza vaccine**												
^b^ Q3. Who are the priority populations that need to take influenza vaccination?												
Healthcare workers	152 (21.9)	417 (60.1)	125 (18)	0.144	216 (31.1)	366 (52.7)	112 (16.1)	<0.001	150 (21.6)	300 (43.2)	244 (35.2)	<0.001
Vulnerable groups and employees	153 (22.8)	390 (58.2)	127 (19)	0.168	220 (32.8)	346 (51.6)	104 (15.5)	0.030	160 (23.9)	280 (41.8)	230 (34.3)	<0.001
People in key places	93 (21.9)	251 (59.2)	80 (18.9)	0.415	120 (28.3)	234 (55.2)	70 (16.5)	0.001	90 (21.2)	185 (43.6)	149 (35.1)	<0.001
Risky populations	169 (22.9)	439 (59.6)	129 (17.5)	0.690	265 (36)	374 (50.7)	98 (13.3)	0.316	192 (26.1)	326 (44.2)	219 (29.7)	0.252
Infants’ caregivers	64 (24.1)	148 (55.6)	54 (20.3)	0.294	85 (32)	133 (50)	48 (18)	0.053	63 (23.7)	113 (42.5)	90 (33.8)	0.072
Pregnant women	29 (22.7)	68 (53.1)	31 (24.2)	0.091	34 (26.6)	67 (52.3)	27 (21.1)	0.014	28 (21.9)	50 (39.1)	50 (39.1)	0.019
^c^ Q4. Do you know how the influenza vaccination frequency is scheduled?												
Once a year	140 (22.4)	380 (60.7)	106 (16.9)	0.324	181 (28.9)	346 (55.3)	99 (15.8)	<0.001	140 (22.4)	259 (41.4)	227 (36.3)	<0.001
Every 5 years	90 (25.8)	195 (55.9)	64 (18.3)		157 (45.0)	157 (45.0)	35 (10.0)		126 (36.1)	170 (48.7)	53 (15.2)	
Have no idea												
^d^ Q5. Do you know when to get the influenza vaccine?												
In autumn and winter	129 (22.7)	344 (60.5)	96 (16.9)	0.536	162 (28.5)	308 (54.1)	99 (17.4)	<0.001	122 (21.4)	244 (42.9)	203 (35.7)	<0.001
All year round	101 (24.9)	231 (56.9)	74 (18.2)		176 (43.3)	195 (48.0)	35 (8.6)		144 (35.5)	185 (45.6)	77 (19.0)	
Have no idea												
^e^ Q6. How long does the flu vaccine protect after a single dose?												
6–8 months	82 (19.3)	267 (63)	75 (17.7)	0.020	106 (25)	231 (54.5)	87 (20.5)	<0.001	74 (17.5)	182 (42.9)	168 (39.6)	<0.001
At least 5 years	148 (26.9)	308 (55.9)	95 (17.2)		232 (42.1)	272 (49.4)	47 (8.5)		192 (34.8)	247 (44.8)	112 (20.3)	
Have no idea												
Q7. Do you know the places where you can get the flu vaccine for seniors?												
Yes	154 (21.3)	449 (62.2)	119 (16.5)	0.002	225 (31.2)	387 (53.6)	110 (15.2)	<0.001	156 (21.6)	321 (44.5)	245 (33.9)	<0.001
No	76 (30.0)	126 (49.8)	51 (20.2)		113 (44.7)	116 (45.8)	24 (9.5)		110 (43.5)	108 (42.7)	35 (13.8)	
Q8. Have you ever heard about influenza vaccination for older adults?												
Yes	167 (22)	458 (60.3)	134 (17.7)	0.089	238 (31.4)	400 (52.7)	121 (15.9)	<0.001	160 (21.1)	344 (45.3)	255 (33.6)	<0.001
No	63 (29.2)	117 (54.2)	36 (16.7)		100 (46.3)	103 (47.7)	13 (6.0)		106 (49.1)	85 (39.4)	25 (11.6)	
Q9. Do you know the policy that older adults aged 60 years and above with Shenzhen household registration could have free influenza vaccination in Shenzhen?												
Yes	163 (22.1)	449 (60.8)	126 (17.1)	0.085	230 (31.2)	392 (53.1)	116 (15.7)	<0.001	155 (21)	332 (45)	251 (34)	<0.001
No	67 (28.3)	126 (53.2)	44 (18.6)		108 (45.6)	111 (46.8)	18 (7.6)		111 (46.8)	97 (40.9)	29 (12.2)	

^a^ *p* value is calculated from a Chi-square test. ^b^ The details of answers for “Q3. Who are the priority populations that need to take influenza vaccination?” are shown below: healthcare workers, including clinicians, public health staff, and health and quarantine staff; vulnerable groups and employees include people in places where people gather, such as elderly care institutions, long-term care institutions, and welfare homes; people in key places include nursery institutions, primary and secondary schools, and prison institutions; risky populations include older people aged 60 and above, children aged 6 months to 5 years, and patients with specific chronic diseases; infants’ caregivers include family members and caregivers of infants aged under 6 months; pregnant women include women who are pregnant or planning to become pregnant during flu season. ^c^ “Once a year” is the correct answer for Q4. ^d^ “In autumn and winter” is the correct answer for Q5. ^e^ “6–8 months” is the correct answer for Q6. ^f^ The number inside of brackets indicates percentages and outside of brackets indicates frequency.

**Table 4:** Attitudes towards the influenza vaccination and education program among older adults (N = 975) in Shenzhen, China.

Items	N	Percentage (%)
Q1. Do you think older adults should take influenza vaccination?		
Yes	861	88.3
No	114	11.7
Q2. Do you think older adults with chronic medical conditions should take influenza vaccination?		
Yes	550	57.1
No	425	43.6
Q3. Do you think participating in a health education program is beneficial for you to learn knowledge about vaccination?		
Yes	652	66.9
No	323	33.1

**Table 5:** Attitudes towards the influenza vaccine and education program in the age, education, and income group analysis (N = 975) in Shenzhen, China ^b^.

Items	Age (Yrs)			^a^ *p* Value	Education Level			^a^ *p* Value	Income Level			^a^ *p* Value
	60~64 (N = 230)	65~75 (N = 575)	Above 75 (N = 170)		Primary and Below (N = 338)	Middle and High School (N = 503)	College and Above (N = 134)		Low (N = 266)	Middle (N = 429)	High (N = 280)	
Q1. Do you think older adults should take influenza vaccination?												
No	28 (24.6)	64 (56.1)	22 (19.3)	0.785	53 (46.5)	51 (44.7)	10 (8.8)	0.013	52 (45.6)	45 (39.5)	17 (14.9)	<0.001
Yes	202 (23.5)	511 (59.3)	148 (17.2)		285 (33.1)	452 (52.5)	124 (14.4)		214 (24.9)	384 (44.6)	263 (30.5)	
Q2. Do you think older adults with chronic medical conditions should take influenza vaccination?												
No	115 (27.1)	251 (59.1)	59 (13.9)	0.010	172 (40.5)	211 (49.6)	42 (9.9)	<0.001	140 (32.9)	195 (45.9)	90 (21.2)	<0.001
Yes	115 (20.9)	324 (58.9)	111 (20.2)		166 (30.2)	292 (53.1)	92 (16.7)		126 (22.9)	234 (42.5)	190 (34.5)	
Q3. Do you think participating in a health education program is beneficial for you to learn knowledge about vaccination?												
No	74 (22.9)	186 (57.6)	63 (19.5)	0.487	137 (42.4)	160 (49.5)	26 (8)	<0.001	134 (41.5)	128 (39.6)	61 (18.9)	<0.001
Yes	156 (23.9)	389 (59.7)	107 (16.4)		201 (30.8)	343 (52.6)	108 (16.6)		132 (20.2)	301 (46.2)	219 (33.6)	

^a^ *p* value is calculated from a Chi-square test. ^b^ The number inside of brackets indicates percentages and outside of brackets indicates frequency.

**Table 6:** Practices of the influenza vaccination among older adults (N = 975) in Shenzhen, China.

Items	N	Percentage (%)
**Cues to actions**		
Q1. Did community health workers recommend the influenza vaccination to you?		
Yes	653	67.0
No	322	33.0
Q2. Are there any older adults around you who had influenza vaccination?		
Yes	580	59.5
No	395	40.5
Q3. Ever participated in a health education activity?		
Yes	333	34.2
No	642	65.8
**Actions**		
Q4. Have you ever received influenza vaccination?		
No, never received	433	44.4
Yes, ever received	542	55.6
Q5. Have you received influenza vaccination in 2020?		
No	521	53.4
Yes	454	46.6
Q6. Reasons for being unwilling or unsure to receive the influenza vaccines		
Stay at a good condition, no need to take vaccine	119	27.5
Do not know about vaccination	186	43.0
Influenza vaccine is not safe enough	28	6.4
Influenza vaccine has no effect	12	2.8
Have no time to take vaccine	54	12.5
Influenza is not important and will not lead to any serious results	30	6.9
It is inconvenient to go to the vaccination places	45	10.4
The cost of vaccination is too much	23	5.3
Physicians do not recommend it	24	5.5
Others	52	12.0

**Table 7:** Practices towards the influenza vaccine in the age, education, and income group analysis (N = 975) in Shenzhen, China ^b^.

Items	Age (Yrs)			^a^ *p* Value	Education Level			^a^ *p* Value	Income Level			^a^ *p* Value
	60–64 (N = 230)	65–75 (N = 575)	Above 75 (N = 170)		Primary and Below (N = 338)	Middle and High School (N = 503)	College and Above (N = 134)		Low (N = 266)	Middle (N = 429)	High (N = 280)	
Q1. Did community health workers recommend the influenza vaccination to you?												
No	87 (27.0)	180 (55.9)	55 (17.1)	0.202	140 (43.5)	156 (48.4)	26 (8.1)	<0.001	130 (40.4)	137 (42.5)	55 (17.1)	<0.001
Yes	143 (21.9)	395 (60.5)	115 (17.6)		198 (30.3)	347 (53.1)	108 (16.5)		136 (20.8)	292 (44.7)	225 (34.5)	
Q2. Are there any older adults around you who had influenza vaccination?												
No	115 (29.1)	212 (53.7)	68 (17.2)	0.003	163 (41.3)	192 (48.6)	40 (10.1)	<0.001	150 (38.0)	171 (43.3)	74 (18.7)	<0.001
Yes	115 (19.8)	363 (62.6)	102 (17.6)		175 (30.2)	311 (53.6)	94 (16.2)		116 (20.0)	258 (44.5)	206 (35.5)	
Q3. Ever participated in a health education activity?												
No	161 (25.1)	364 (56.7)	117 (18.2)	0.130	254 (39.6)	306 (47.7)	82 (12.8)	<0.001	217 (33.8)	265 (41.3)	160 (24.9)	<0.001
Yes	69 (20.7)	211 (63.4)	53 (15.9)		84 (25.2)	197 (59.2)	52 (15.6)		49 (14.7)	164 (49.2)	120 (36.0)	
Q4. Have you ever received influenza vaccination?												
No	130 (30.0)	238 (55.0)	65 (15.0)	<0.001	186 (43.0)	209 (48.3)	38 (8.8)	<0.001	166 (38.3)	200 (46.2)	67 (15.5)	<0.001
Yes	100 (18.5)	337 (62.2)	105 (19.4)		152 (28.0)	294 (54.2)	96 (17.7)		100 (18.5)	229 (42.3)	213 (39.3)	
Q5. Have you received influenza vaccination in 2020?												
No	142 (27.3)	298 (57.2)	81 (15.5)	0.010	212 (40.7)	252 (48.4)	57 (10.9)	<0.001	179 (34.4)	235 (45.1)	107 (20.5)	<0.001
Yes	88 (19.4)	277 (61.0)	89 (19.6)		126 (27.8)	251 (55.3)	77 (17.0)		87 (19.2)	194 (42.7)	173 (38.1)	

^a^ *p* value is calculated from a Chi-square test. ^b^ The number inside of brackets indicates percentages and outside of brackets indicates frequency.

**Table 8:** Multivariate regression analysis of the associated factors of covariates on the previous actions of influenza vaccination and actions.

Factors	Actions of Vaccination				Actions of Vaccination in 2020			
	Never Received N (%)	Ever Received N (%)	^a^ *p* Value	OR (95% CI)	Not Received N (%)	Received N (%)	*p* Value	OR (95% CI)
Sex								
Female	239 (43.5)	310 (56.5)	0.532		290 (52.8)	259 (47.2)	0.663	
Male	194 (45.5)	232 (54.5)			231 (54.2)	195 (45.8)		
Age (yrs)								
60–64	130 (56.5)	100 (43.5)	<0.001	ref	142 (61.7)	88 (38.3)	0.010	ref
65–75	238 (41.4)	337 (58.6)		1.46 (1.01, 2.10)	298 (51.8)	277 (48.2)		1.18 (0.83, 1.68)
Above 75	65 (38.2)	105 (61.8)		1.81 (1.10, 2.98)	81 (47.6)	89 (52.4)		1.80 (1.11, 2.92)
Marital status								
Unmarried	86 (56.6)	66 (43.4)	0.001	ref	103 (67.8)	49 (32.2)	<0.001	ref
Married	347 (42.2)	476 (57.8)		1.18 (0.77, 1.84)	418 (50.8)	405 (49.2)		1.54 (0.99, 2.36)
Education level								
Primary school and below	186 (55.0)	152 (45.0)	<0.001	ref	212 (62.7)	126 (37.3)	<0.001	ref
Middle and high school	209 (41.6)	294 (58.4)		1.12 (0.79, 1.60)	252 (50.1)	251 (49.9)		1.14 (0.81, 1.60)
College and above	38 (28.4)	96 (71.6)		1.12 (0.64, 1.96)	57 (42.5)	77 (57.5)		0.94 (0.57, 1.56)
Monthly income (CNY)								
Low (1000 and below)	166 (62.4)	100 (37.6)	<0.001	ref	179 (67.3)	87 (32.7)	<0.001	ref
Middle (1001–5000)	200 (46.6)	229 (53.4)		1.32 (0.90, 1.92)	235 (54.8)	194 (45.2)		1.19 (0.82, 1.73)
High (5001 and above)	67 (23.9)	213 (76.1)		2.64 (1.66, 4.18)	107 (38.2)	173 (61.8)		1.69 (1.10, 2.61)
Self-reported health status								
Well	265 (44.8)	327 (55.2)	0.442		306 (51.7)	286 (48.3)	0.197	
General	134 (42.4)	182 (57.6)			173 (54.7)	143 (45.3)		
Worse	34 (50.7)	33 (49.3)			42 (62.7)	25 (37.3)		
Chronic medical conditions								
No	223 (41.8)	311 (58.2)	0.067		240 (54.4)	201 (45.6)	0.575	
Yes	210 (47.6)	231 (52.4)			281 (52.6)	253 (47.4)		
Received COVID-19 vaccine uptakes								
No	83 (51.2)	79 (48.8)	0.056		106 (65.4)	56 (34.6)	0.001	ref
Yes	350 (43.1)	463 (56.9)			415 (51.0)	398 (49.0)		1.57 (1.05, 2.37)
How long does the flu vaccine protect after a single dose?								
At least 5 years	336 (61.0)	215 (39.0)	<0.001	ref	372 (67.5)	179 (32.5)	<0.001	ref
Have no idea								
6–8 months	97 (22.9)	327 (77.1)		2.76 (1.99, 3.84)	149 (35.1)	275 (64.9)		2.15 (1.58, 2.93)
Do you know where you can get the flu vaccine for seniors?								
No	203 (80.2)	50 (19.8)	<0.001	ref	309 (42.8)	413 (57.2)	<0.001	ref
Yes	230 (31.9)	492 (68.1)		5.00 (3.42, 7.32)	212 (82.8)	41 (16.2)		4.22 (2.85, 6.24)
Do you think older adults with chronic medical conditions should take influenza vaccination?								
No	267 (62.8)	158 (37.2)	<0.001	ref	294 (69.2)	131 (30.8)	<0.001	ref
Yes	166 (30.2)	384 (69.8)		2.08 (1.51, 2.85)	227 (41.3)	323 (58.7)		1.73 (1.27, 2.35)

^a^ *p* value is calculated from multivariable regression analysis.

## Data Availability

Data will be made available on request.
